# Pitch and Timbre Interfere When Both Are Parametrically Varied

**DOI:** 10.1371/journal.pone.0087065

**Published:** 2014-01-21

**Authors:** Valeria C. Caruso, Evan Balaban

**Affiliations:** 1 Cognitive Neuroscience Sector, SISSA, Trieste, Italy; 2 Behavioral Neurosciences Program, McGill University, Montreal, QC, Canada; 3 Center for Cognitive Neuroscience, Duke University, Durham, North Carolina, USA; University of Chicago, United States of America

## Abstract

Pitch and timbre perception are both based on the frequency content of sound, but previous perceptual experiments have disagreed about whether these two dimensions are processed independently from each other. We tested the interaction of pitch and timbre variations using sequential comparisons of sound pairs. Listeners judged whether two sequential sounds were identical along the dimension of either pitch or timbre, while the perceptual distances along both dimensions were parametrically manipulated. Pitch and timbre variations perceptually interfered with each other and the degree of interference was modulated by the magnitude of changes along the un-attended dimension. These results show that pitch and timbre are not orthogonal to each other when both are assessed with parametrically controlled variations.

## Introduction

In everyday life, people attend simultaneously to both the pitch and timbre of sounds. In speech, the pitch contour of a sentence carries prosodic information, while timbre variations enable listeners to identify phonemes and vowels necessary for speech segmentation; both contribute to speaker gender and body size perception [1]. Both are also central to music perception: pitch typically defines melodies and timbre distinguishes different instruments.

Pitch and timbre have been historically viewed as orthogonal perceptual dimensions [2], but they both are based on spectro-temporal frequency information. Previous studies have produced mixed results on whether these dimensions interfere with each other or not, and thus on whether they may be processed entirely independently [3]–[19].

We hypothesized that these discrepant results might relate to unequal manipulation of the perceptual spacing of pitch and timbre cues, as well as to variations in subjects’ previous experience with particular timbres, especially musical timbres. Thus, we sought to assess the interactions between pitch and timbre by parametrically varying them in the threshold-to-supra-threshold range using novel sounds. Listeners attended to one dimension at a time, and judged whether two sounds presented in sequence differed along the attended dimension. Such sequential comparisons are ecologically relevant because meaningful pitch and timbre variations in music and speech streams typically involve serial comparisons over time.

## Methods

### Stimuli

Sounds were synthesized using SIGNAL, a Digital Programming Language (version 5.04.17, Engineering Design, Berkeley, CA). We filtered white noise with a spectrum made up from the sum of 10 Gaussian filters (Equation 1) with constant width and exponentially decaying amplitude, centered at the first 10 harmonics of a fundamental frequency f_0_.
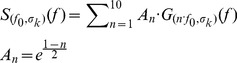
(1)


In Equation 1, S is the filtering spectrum, G is an individual Gaussian filter (with unitary amplitude, peak at the central frequency n•f_0_ and width σ_k_) and A is the exponentially decaying weight scaling each Gaussian filter (10 filters in the spectrum, indicated by the index n).

Sounds with different timbres were characterized by differences in their filter width (σ_k_ in Equation 1), and sounds with different pitches were characterized by their fundamental frequency f_0_. Two independent experiments, a timbre-rating test (see Timbral step spacing determination below and Figure S1) and a pitch-matching test (Results), proved that filter width variation was related to the perceived timbre (on a continuum from tonal to noise-like) and fundamental frequency variation was related to the perceived pitch of the sounds.

The timbre rating experiment also provided a metric for selecting a set of sounds with different filter widths that were evenly spaced along the timbre dimension and easily discriminable by all subjects. The selected filter widths were σ_k_ = 10, 16.9, 25.1, 32.6, 48 Hz, corresponding to sounds defined, for the purposes of this experiment, as one “timbral step” apart. The fundamental frequencies were f_0_ = 300, 318, 337, 357, 378 Hz, corresponding to sounds one semitone (one experimental “pitch step”) apart. No rating procedure was required for pitch, because it is known that its perception depends on log-linear frequency ratios between stimuli [20]. It was also not important for the design of this experiment whether timbre steps were of the same “perceptual size” as pitch steps: it was only necessary to ensure that, within each dimension, a perceptually-consistent step size was used.

Two sets of sounds were prepared for the two conditions of the task (described below).

For the attend-timbre condition we chose 4 filter widths (σ_k_ = 16.9, 25.1, 32.6, 48 Hz) and 3 fundamental frequencies (f_0_ = 300, 318, 378 Hz). These values yielded 4 intra-pair timbre distances: 0 timbral steps (same σ_k_ for the two sounds), 1 timbral step (pairs with adjacent values of σ_k_ in the above list, for example 16.9 and 25.1), 2 timbral steps (pairs with σ_k_ values two steps apart, for example 16.9 and 32.6), 3 timbral steps (pairs with σ_k_ values 3 steps apart, 16.9 and 48); as well as 4 intra-pair pitch distances: 0 semitones (same f_0_ for the 2 sounds), 1 semitone (pairs with f_0_ = 300 and f_0_ = 318), 3 semitones (pairs with f_0_ = 318 and f_0_ = 378), and 4 semitones (pairs with f_0_ = 300 and f_0_ = 378).

For the attend-pitch condition, we chose 4 fundamental frequencies (f_0_ = 318, 337, 357, 378 Hz) and 3 filter widths (σ_k_ = 16.9, 25.1, 48 Hz), yielding, in the same way as described in the preceding paragraph, 4 intra-pair pitch distances (0, 1, 2, 3 semitones) and 4 intra-pair timbre distances (0, 1, 2, 3 timbral steps).

**Figure 1:**
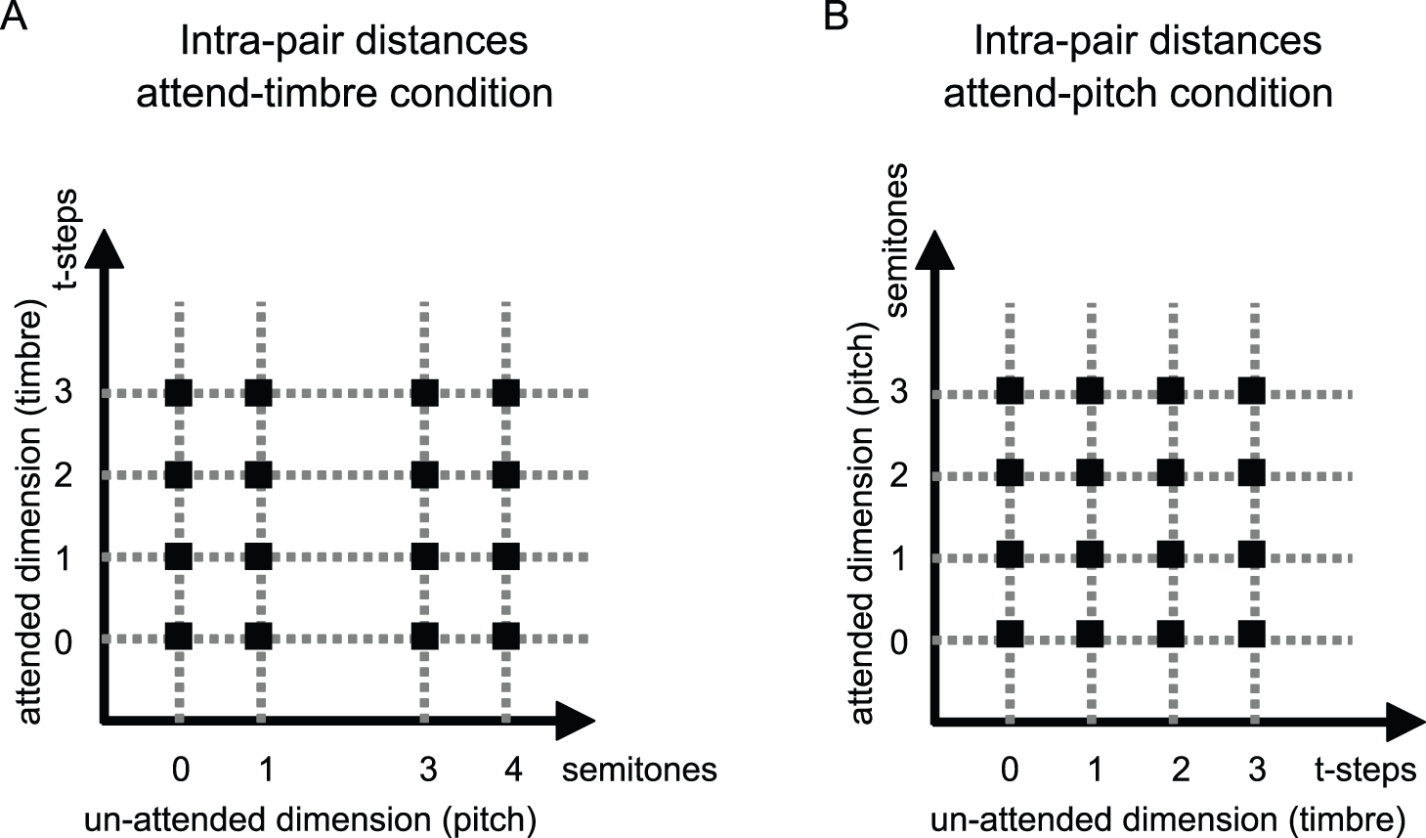
Intra-pair pitch and timbre distances. (A) attend-timbre condition, (B) attend-pitch condition. For each pair of sounds the timbre and pitch variations are indicated in the graphs. For each single sound in the pairs, pitch is related to the fundamental frequency, f_0_, and timbre is related to the width of the Gaussian filter in the spectrum, σ_k_ (Equation1). The intra-pair distances in (A), attend-timbre condition, are obtained with sounds parameters: f_0_ = 300, 318, 378 Hz, σ_k_ = 16.9, 25.1, 32.6, 48 Hz. The intra-pair distances in (B), attend-pitch condition, are obtained with sounds parameters: f_0_ = 318, 337, 357, 378 Hz, σ_k_ = 16.9, 25.1, 48 Hz.

Thus, for each stimulus set, the dimension relevant for the task (pitch or timbre intra-pair distance) could take 4 levels, while the irrelevant dimension could take 3 levels (Figure 1). All sounds were 500 ms long, including a 50 ms cosine rise and fall. The interval between sounds within a pair was 300 ms.

To obtain the same loudness, the sounds were equated for their RMS value [21].

### Timbral Step Spacing Determination

A dissimilarity rating task was used to test how naïve listeners perceived the timbre of the experimental sound stimuli at a constant fundamental frequency. The goal was to adjust the width of the Gaussian filters in the sound spectrum (σ_k_, Equation 1), in order to obtain a set of stimuli evenly spaced along the timbre dimension.

Ten sounds were synthesized as described above, with constant fundamental frequency (f_0_ = 300 Hz), duration (500 ms) and loudness (equal RMS; [21]) and ten levels of timbre (σ_k = _10, 15, 20, 25, 30, 35, 40, 45, 50, 55, Equation 1). They were arranged in all possible pairs with 300 ms inter-sound intervals.

Eight subjects (2 men, age range 20–35 years, volunteers) gave written informed consent and reported having no hearing deficits. They were paid 8 €. The subjects first listened to all 10 sounds, presented singly for familiarization. Then they listened to each pair of sounds in random order (6 repetitions) and rated the dissimilarity of the sounds in the pair on a scale from 0 to 9, where 0 meant “identical” and 9 meant “very different”. They were not instructed about, and not asked to focus on, the way in which the sounds varied. Each pair could be heard more than once, with no time limit.

These data were analyzed using custom-written programs in MATLAB R2007a (Version 7.4.0.287, The MathWorks, Natick MA). For each subject, the average over six ratings of the same pair was calculated (pairs made up of the same sounds in a different order counted as the same pair). A linear regression analysis of the average ratings on the timbre intra-pair distances was then computed. Data from one subject were excluded at this stage, because his ratings did not appear to scale with intra-pair distance, as indicated by the squared correlation coefficient (r^2^) between this subject’s rating and the parametric intra-pair distances (r^2^ = 0.21). All the other subjects (N = 7) gave ratings that scaled with the parametric intra-pair distances (r^2^>0.63) and were in strong agreement with each other (the average of the inter-subjects squared correlation coefficients was 0.70).

The judgments of the remaining subjects (N = 7) were averaged and rearranged into a 10×10 upper triangular matrix with no diagonal. Multidimensional scaling [30] was used to reconstruct, from this dissimilarity matrix, a map of the position of the sounds in an acoustic space that could best account for the perceived distances. Since the response scale used here only assumed an ordinal scale of measurement, we chose a non-metric MDS algorithm (Matlab function mdscale: nonmetric scaling with Kruskal's nonmetric stress criterion). We used the values of stress associated with the MDS results to evaluate how well a particular configuration reproduced the observed distance matrix. The MDS analysis suggested that the timbre of the tested sounds was best described by a 2 dimensional space, although 1 dimension was already a good approximation (the stress was 0.026 for 1 dimension and less than 0.001 for 3 dimensions, Figure S1).

These results provided a quantitative representation of the sounds that approximated the ranks of the dissimilarities. As all sounds were equated for duration, intensity and pitch, it was assumed that the dimensions found by the MDS algorithm were inherent to timbre. The goal of this analysis was not to try to interpret what acoustic variables contributed to timbre, so that we did not systematically attempt to determine what acoustic qualities were represented by the dimensions produce by the MDS analysis. Rather, the purpose was to have a systematic, albeit approximate, way of reorganizing the parameters of the sound-generating function in order to produce a set of stimuli that a typical human subject would perceive as evenly-spaced along the timbre dimension. Thus, the MDS solution in the three dimensional space was used to adjust the parameters of the sound-generating function, and obtain a new evenly-spaced timbre series.

In the new series, distances between adjacent parameters σ_k_ were proportional to the inverse of the distances between adjacent sounds, as reconstructed by the MDS algorithm. The new series was σ_k = _10, 13.5, 16.9, 21.0, 25.1, 28.5, 32.6, 39.5, 48.0, 55 Hz. To have adjacent sounds perceptually distinguishable, only five levels were used in the main experiment: σ_k = _10, 16.9, 25.1, 32.6, 48.0 Hz.

### Procedures

Subjects heard pairs of sounds (binaurally through Sennheiser HD 580 headphones at a constant level of their choice) and indicated whether the pairs were the same or different along the specified dimension –timbre for the first condition (attend-timbre) or pitch for the second condition (attend-pitch)– by pressing a key on the keyboard with no time limit. The sounds in each pair could vary along both dimensions (Figure 1).

Both conditions included an initial training phase to familiarize subjects with the task and the terminology, and to select a homogeneous group of participants: those who reached a minimum performance criterion (described below) were admitted to the test phase.

During training, subjects received feedback after judging each pair, and repeated trials in which they made the wrong judgment. After five consecutive correct answers, the subjects took a small exam with no feedback: they judged 10 pairs, from among the most difficult pairs (having close or equal values along the relevant dimension). The criterion to be admitted to the test phase was a score of 7 or more correct answers in this exam.

In the test phase all the possible pairs were presented 6 times in random order, with no feedback.

### Subjects

All procedures were approved by the SISSA Ethics Committee. All participants (75 total, 24 men, age range 18–35 years) gave written informed consent and reported having no hearing deficits. They were paid 8€ per hour.

All 75 subjects were tested in the attend-timbre condition in the initial session of these experiments; 57 subjects (21 men) successfully passed the training phase and completed the task. The attend-pitch condition was tested during a second, later session. A subset of 21 subjects (6 men) who had successfully completed the attend-timbre condition was selected blindly with respect to their performance in the attend-timbre condition. They all successfully completed the training session and the task. A minimum of 2 days intervened between the two conditions for each subject.

### Analysis

All analyses were performed using custom-written programs in MATLAB R2007a (Version 7.4.0.287, The MathWorks, Natick MA).

For each participant, the hit and false alarm rates were computed (proportions of correct and incorrect “different” responses, respectively) for each intra-pair distance along the attended and un-attended dimension in the test phase (6 repetitions in each condition). Average values are shown in Table 1.

**Table 1:** Mean hit rate and false alarm rates for all timbre and pitch distances in the two conditions.

Condition	Distances along the unattended dimension	Hits	FA 1 t-step	FA 2 t-steps	FA 3 t-steps
Attend-timbre	Same distance	0.96 (0.01)	0.57 (0.02)	0.09 (0.02)	0.03 (0.01)
	1 semitone apart	0.86 (0.02)	0.44 (0.02)	0.07 (0.01)	0.03 (0.01)
	3 semitone apart	0.71 (0.03)	0.41 (0.02)	0.10 (0.02)	0.01 (0.004)
	4 semitone apart	0.63 (0.03)	0.37 (0.02)	0.09 (0.01)	0.02 (0.01)
Attend-pitch	Same distance	0.98 (0.01)	0.08 (0.02)	0.02 (0.06)	0.01 (0.07)
	1 t-step apart	0.95 (0.04)	0.05 (0.02)	0.02 (0.03)	0.02 (0.03)
	2 t-steps apart	0.81 (0.01)	0.10 (0.02)	0.05 (0.02)	0.02 (0.03)
	3 t-steps apart	0.75 (0.004)	0.10 (0.01)	0.07 (0.01)	0.02 (0.01)

(SE in parentheses).

**Figure 2:**
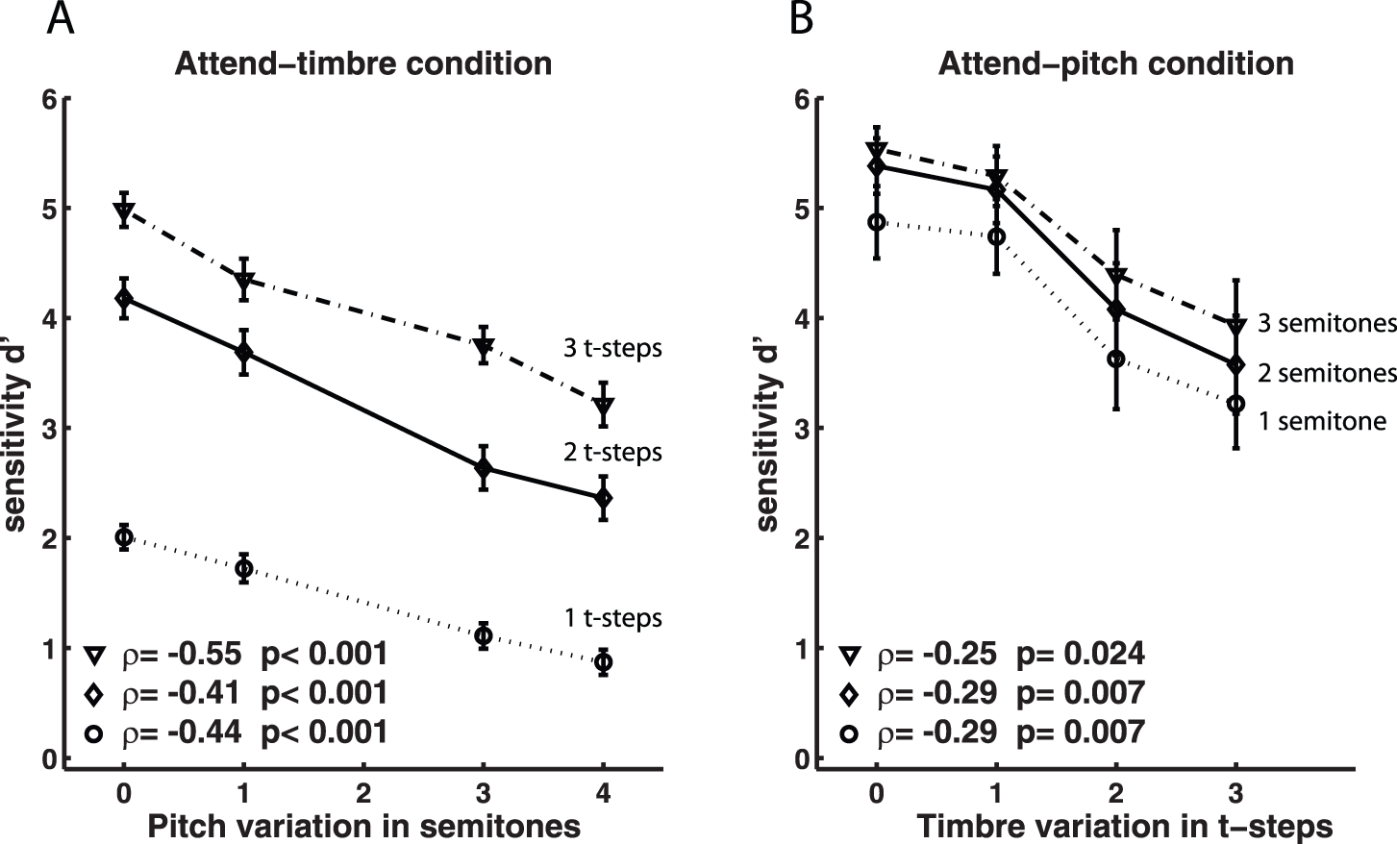
Average sensitivity as a function of pitch and timbre variations. Values represent average d’ score (±SE) across subjects, for each intra-pair distance along the attended dimension (1step circle, 2 steps diamond or 3 steps triangle) and un-attended dimension (on the x-axis). (A) attend-timbre condition, n = 57 for each data point; (B) attend-pitch condition, n = 21 for each data point. ρ indicates the Spearman rank correlations coefficient between the d’ scores and the variations along the un-attended dimension, p indicates the p-values for the significance test.

We computed the d’ scores of individual participants from the hit and false alarm rates [22]. d’ scores measure discriminability, the separation between the means of the signal and the noise distributions, in units of the standard deviation of the noise distribution. A d’ score equal to zero indicates that subjects are unable to discriminate between change and no change; a d’ score significantly greater than zero means that subjects can detect the difference between change and no change. Once d’ scores are significantly different from zero, higher d’ scores indicate that a difference is more readily perceived. Group d’ scores are shown in Figure 2.

Non-parametric statistical analyses were carried out on the d’ scores, as the normality assumption was violated for most combinations of timbre and pitch distances (Lilliefors tests, Table 2).

**Table 2:** Lilliefors normality test p-values for the d’ scores in all conditions.

Condition	Distances along theunattended dimension	1 step along theattended dimension	2 steps along theattended dimension	3 steps along theattended dimension
Attend-timbre	Same distance	0.500	0.055	0.001*
	1 semitone apart	0.013*	0.113	0.005*
	3 semitone apart	0.005*	0.351	0.038*
	4 semitone apart	0.016*	0.222	0.088
Attend-pitch	Same distance	0.003*	0.001*	0.001*
	1 t-step apart	0.001*	0.001*	0.001*
	2 t-steps apart	0.088	0.032*	0.001*
	3 t-steps apart	0.349	0.183	0.365

* p-values <0.05 indicate a non-normal distribution.

Wilcoxon signed-rank tests [23] were used for assessing whether the d’ scores were significantly different from zero in all combinations of timbre and pitch distances.

The interaction between pitch and timbre was assessed separately in the attend-timbre and attend-pitch conditions by the Scheirer-Ray-Hare extension of the Kruskal-Wallis test ([24], a non-parametric two-way ANOVA, factors: distance along the attended and un-attended dimension).

Nonparametric Spearman rank correlations [23] were used for calculating correlation coefficients between the d’ scores and the variations along the un-attended dimension (Figure 2) and a non-parametric ANCOVA based on ranks [25] was used for testing the hypotheses of equal slopes and intercepts.

## Results

Figure 2 shows the average d’ (mean±SE) across subjects (57 subjects in the attend-timbre condition and 21 subjects in the attend-pitch conditions), obtained from the answers given to identical and different stimulus pairs along the attended dimension. In all conditions, the discriminability significantly decreased as the variation in the un-attended dimension grew larger, indicating interference between pitch and timbre (Spearman rank correlations were all negative and significantly different from zero, indicated in Figure 2). Nevertheless, a degree of distinction between timbre and pitch was retained under all interference conditions: the ability of subjects to detect change at significantly-greater-than chance levels was never abolished by variations along the irrelevant dimension (the d’ scores in all conditions remained significantly different from zero - Wilcoxon signed-rank tests, all p<0.001). In other words, the variations along the irrelevant dimension never fully determined the subjects’ response to the pairs. Significant interference between pitch and timbre, which did not disrupt the subjects’ ability to perform the task, was confirmed by nonparametric two-way analysis of variance [24] and rank analysis of covariance [25]; both analyses indicated that the interference was similar for different distances along the attended dimensions.

The Scheirer-Ray-Hare extension of the Kruskal-Wallis test [24], a nonparametric two-way analysis of variance, yielded a main effect for the variations along the attended dimension of timbre (attend-timbre: H(2) = 275.64, p<0.001) but not along the attended dimension of pitch (attend-pitch: H(2) = 5.30, p = 0.07) indicating that in the attend-timbre condition, an increase in the timbre distance yielded a substantial increase in discriminability, while in the attend-pitch condition, discriminability was roughly equivalent at all 3 pitch distances (a “ceiling” effect due to the fact that a semitone is already an easily discriminable pitch interval). The main effect of the un-attended dimension was significant in both conditions (attend-timbre: H(3) = 82.89, p<0.001; attend-pitch: H(3) = 33.11, p<0.001), indicating a clear interference between pitch and timbre both when subjects judged timbre and when they judged pitch. The interaction term was not significant (attend-timbre: H(6) = 3.31, p = 0.77; attend-pitch: H(6) = 0.14, p = 0.99), indicating that the effect of variations along the un-attended dimension were consistent across variations along the attended dimension.

The rank analysis of covariance [25] confirmed these results in both attend-timbre and attend-pitch conditions. We considered the ranked d’ values, and tested the linear regression model of 3 lines with equal slopes and different intercepts against the model with different slopes and different intercepts (test one). Since the model with equal slopes and different intercepts was not rejected in both conditions (see below), we tested it against the model with equal slopes and equal intercepts (test two).

In the attend-timbre condition, the first test showed no indication of statistically different slopes (F(2,678) = 1.82, p = 0.163), while the second test showed statistically different intercepts (F(2,680) = 76.42, p<0.001). In other words, 3 different but parallel lines fit the ranked d’ values at variable timbre distances. This confirms that an increase in the timbre distance yielded a substantial increase in discriminability, and that variations along the unattended dimension of pitch affected discriminability similarly for all timbre distances.

In the attend-pitch condition, the results for the two tests showed no indication of statistically different slopes (F(2,246) = 1.04, p = 0.353) or intercepts (F(2,248) = 1.36, p = 0.259). In other words, the same line fits the ranked d’ values at variable pitch distances. This confirms that performance for different pitch distances was at “ceiling”, but nevertheless, variations along the unattended dimension of timbre affected pitch discriminability.

The “step sizes” for pitch and timbre used in this experiment were perceptually different (the smallest timbre distance was less discriminable than the smallest pitch distance, according Wilcoxon rank sum tests, all of which yielded p<0.001). Indeed, all discriminations in the attend-pitch condition were easier for subjects to perform than in the attend-timbre condition (Figure 2). On average, in the attend-pitch condition subjects also completed the training phase faster (26.33 vs. 94.71 average trials; Wilcoxon matched pair sign rank test: z = −4.02, p<0.0001).

To ascertain whether the timbre manipulation used here may have inadvertently produced small but robust directional changes in pitch percepts, a frequency-matching control experiment was conducted. The experimental set-up was the same as the main experiment. Ten subjects (9 women, age range: 20–35 years) were selected from a pool of 15 new volunteers using the same performance criteria as the main experiment. They listened to each sound from the experimental set (5 repetitions, random order) and adjusted the frequency of a pure tone to match it. No time limit was set, and no feedback was given to the subjects about their performance. Four levels of pitch (f_0_ = 300, 318, 357, 378) and four levels of timbre (σ_k_ = 16.9, 25.1, 32.6 48) were used. For each individual and for each pitch level, the matched tone frequencies were regressed against the timbre levels. There were no consistent directional effects of timbre variations on pitch perception (the slopes of the regression lines were not significantly different from zero, Table 3). The variability of the matched tone frequencies (measured as the standard deviation of the matched values for each subject and each target sound) were similarly analyzed using regression (Table 4). Again, there were no consistent effects of variability in the timbre dimension on the variability of pitch percepts. This failure of pitch percepts to appear more difficult as filter width increased was also confirmed by a similar analysis on the number of adjustments taken for each stimulus which also did not reveal any significant variation that depended on timbre variation (data not shown). These results are consistent with previous studies [5], [18].

**Table 3:** No consistent directional effects of timbre on pure tone matching.

Subject	f_0_ = 300 Hz	f_0_ = 318 Hz	f_0_ = 357 Hz	f_0_ = 378 Hz
S1	0.02*	0.76	0.27	0.82
S2	0.17	0.71	0.14	0.57
S3	0.09	0.15	0.97	0.08
S4	0.47	0.03*	0.03*	0.14
S5	0.57	0.09	0.26	0.15
S6	0.02*	0.12	0.58	0.08
S7	0.31	0.19	0.57	0.31
S8	0.56	0.92	0.16	0.05*
S9	0.10	0.42	0.01**	0.01**
S10	0.24	0.86	0.98	0.75

**Note:** The table shows uncorrected p-values for the slope of individual regression lines (matched tones regressed on timbre levels for each fundamental frequency f_0_). p-values >0.05 mean that the hypothesis of equal slopes cannot be rejected. Uncorrected p-values are shown in this instance because these are the more conservative alternative when making the argument of no consistent directional effects. S1–10 indicates subjects from 1 to 10.

* uncorrected p-value <0.05.

** uncorrected p-value <0.01.

**Table 4:** No consistent effect of timbre on the variability of pure tone matching.

Subject	f_0_ = 300 Hz	f_0_ = 318 Hz	f_0_ = 357 Hz	f_0_ = 378 Hz
S1	0.71	0.38	0.37	0.94
S2	0.33	0.77	0.16	0.39
S3	0.54	0.32	0.07	0.62
S4	0.52	0.83	0.51	0.07
S5	0.91	0.29	0.83	0.05*
S6	0.23	0.20	0.09	0.60
S7	0.26	0.46	0.58	0.43
S8	0.74	0.79	0.06	0.13
S9	0.70	0.40	0.02*	0.41
S10	0.47	0.88	0.75	0.53

**Note:** The table shows uncorrected p-values for the slopes of the individual regressions of the standard deviation of the matched tones on timbre levels for each fundamental frequency f_0_. Uncorrected p-values are shown in this instance because these are the more conservative alternative when making the argument of no consistent directional effects. p-values >0.05 mean that the hypothesis of equal slopes cannot be rejected. S1–10 indicates subjects from 1 to 10.

* uncorrected p-value <0.05.

## Discussion

A sequential comparison task revealed a consistent interference between pitch and timbre variations for a set of novel sounds, where pitch and timbre were both appropriately quantified and parametrically varied. A group of listeners judged whether two sounds presented in sequence differed along one attended dimension (pitch or timbre) while the second dimension varied in an uncorrelated way. The listeners were always able to perform the task at above-chance levels, but had their judgments consistently biased by variations along the unattended dimension. The possibility that the timbre manipulation procedure may have inadvertently produced pitch changes was empirically ruled out by a frequency-matching control experiment.

Alternatively, interactions between pitch and timbre may also be caused by poor selective attention or by a misunderstanding of pitch and timbre concepts on the part of listeners. We believe this was not the case in the present experiments, because of the performance criterion that participants had to meet in order to be included. Indeed, the irrelevant variations never completely dominated the response patterns of subjects: even large pitch variations when the timbre was kept constant did not lead to a complete deterioration of timbre discrimination, indicating that throughout the experiment, the subjects were following instructions and the conceptual distinction between timbre and pitch was retained even under strong interference conditions.

Our results both explain and extend the inconsistent pattern of findings from previous studies using salient timbre manipulations that were not parametrically or perceptually scaled [3], [4], [13], [14], [16]–[19] (but see [15]). These studies mostly focused on musical timbre (recorded or synthesized instrumental sounds) or on brightness, which is one of the major aspects of musical timbre. In some of these studies, salient variations along the irrelevant dimension (instrument identity for timbre or different octaves for pitch) consistently interfered with small variations in the relevant dimension for both pitch and timbre, e.g. [3], [13], [16] but the interaction was inconsistent for larger variations along the relevant dimension [13], [14], [18], often depending on the musical training of the subjects (which in turn correlated with perceptual acuity). Unfortunately, because previous studies did not parametrically measure or control the amount of timbre variation they imposed on their stimuli, it is very difficult to meaningfully compare the relative effect sizes seen in previous work with what was found here.

The present study found a symmetrical interaction between pitch and timbre for controlled perceptual step sizes. The timbre variations were supra-threshold but were relatively small in comparison to the typical variations adopted in previous studies. These sounds were reported by listeners to vary along the continuum between noisy and tonal, rather than being categorically different (such as a trumpet vs. a piano). A symmetrical interaction was also previously observed in long sound sequences [6], [16] and in single-sound speeded classification tasks (but only for the speed, and not the accuracy of judgements [7]–[9], [12], [14]).

The sounds created for this study share similarities with iterated rippled noise (IRN). IRN is constructed by adding a random noise to a copy of itself that is delayed (with a delay d) and attenuated (with a scale factor g) for a number of iterations (n). It corresponds to the environmental case of a sound mixed with its multiple reflections from regularly spaced flat surfaces [26]. Since IRN has temporal regularities, it possesses a pitch at the reciprocal of the delay d, and a timbre (described variously as tone/noise ratio, pitch strength, or pitch saliency) related to the number of iterations n (more iterations produce a more tonal sound, i.e. a more salient pitch) and to the attenuation g (a bigger attenuation generates a stronger noise percept, and a less salient pitch [27], [28]). These pitch and timbre attributes can be extracted from the sound spectrum or from the autocorrelation function (which is the Fourier transform of the power spectrum [28], [29]).

While the sounds in this study share some of the perceptual attributes of IRN, in that both have a pitch that varies in strength from a noisy to a tonal quality, and both have similar spectra and autocorrelation functions, the sounds used in this study differ from IRN in that the amplitude of their spectral peaks decays exponentially from low frequencies to high frequencies. This minimizes any conflict between spectral and periodicity pitch cues. While the pitch and pitch saliency ( = timbre) of IRN stimuli have been considered as independent percepts [26], to our knowledge there is no previous study that has examined the perceptual interaction between pitch and timbre in these sounds. Such testing could reveal a similar effect to the one found here, where, in spite of a clear pitch and identifiable timbral quality of particular sounds, variations along one of the dimensions may become less identifiable when the second dimension also varies.

Interference between pitch and timbre has been interpreted as a conflict based on common mechanisms used for “spectral” contributions to pitch and timbre determination [5], [6], [9], [13], [18], [19]. In pitch perception, spectral information is presumably combined with periodicity information. The timbre manipulation used in this study minimizes the conflict between spectral and periodicity pitch, since the fundamental always has the maximum amplitude. Future experiments examining pitch-timbre interference at small perceptual distances for sounds where spectral and temporal mechanisms make unequal contributions to pitch perception could provide a fuller understanding of the joint contributions of spectral and temporal information to both pitch and timbre processing.

In summary, a consistent interference between pitch and timbre can be identified when timbre and pitch are appropriately quantified and parametrically varied. The interference does not abolish the distinction between pitch and timbre, but variations along the un-attended dimension make listeners less certain about whether they heard variation in the attended dimension.

## Supporting Information

Figure S1 **Results of the multidimensional scaling (MDS).** (a) Stress as a function of the dimensionality of the MDS solution; (b) three-dimensional spatial representation based on the dissimilarity ratings for 10 sounds.
(EPS)
